# Chemical Changes in Deep‐Fat Frying: Reaction Mechanisms, Oil Degradation, and Health Implications

**DOI:** 10.1002/fsn3.70969

**Published:** 2025-10-13

**Authors:** Naser Bazina, Tariq Ahmed, Mostafa Almdaaf, Husayn Mohammed Omar Abu Hallalah, Shamsudeen Jibia

**Affiliations:** ^1^ School of Health and Life Sciences Teesside University Middlesbrough UK; ^2^ Libyan Biotechnology Research Centre Tripoli Libya; ^3^ School of Computing Engineering and Digital Technologies Teesside University Middlesbrough UK; ^4^ Department of Medicinal Chemistry, Faculty of Pharmacy Elmergib University Alkhoms Libya; ^5^ Umar Musa Yar'adua University Katsina Katsina Nigeria

**Keywords:** food quality, food safety, lipid oxidation, polymerization reactions, thermal stability

## Abstract

Deep‐fat frying remains a prevalent culinary technique due to its ability to enhance sensory properties; however, it also initiates complex physicochemical transformations that compromise oil integrity and food safety. This review critically evaluates the current understanding of thermal degradation pathways in frying oils, including oxidation, hydrolysis, and polymerization, highlighting the underlying reaction mechanisms and their interdependence. The influence of intrinsic oil properties (e.g., fatty acid composition, minor components) and extrinsic factors (e.g., temperature, food matrix, fryer design, metal contaminants) on oxidative stability is assessed with particular attention to inconsistencies in reported degradation kinetics. Comparative analysis of conventional versus advanced analytical methods (e.g., titration vs. GC, HPLC, BBCEAS) underscores the limitations of legacy approaches and advocates for the adoption of more sensitive, specific techniques. Furthermore, the review contrasts the efficacy and thermal stability of synthetic and natural antioxidants, while examining conflicting findings on replenishment practices and the impact of emerging technologies such as air and vacuum frying. The health implications of degradation products are critically appraised, including evidence linking frying by‐products to chronic diseases. Finally, gaps in the literature are identified, including the need for mechanistic studies under real frying conditions and standardized assessment protocols. Future research should prioritize oil formulation, frying system design, and regulation to ensure food safety and public health.

## Graphical abstract

This review critically examines the chemical degradation of frying oils, focusing on oxidation, hydrolysis, and polymerization mechanisms and their influencing factors. It highlights limitations in conventional analysis, explores the stability of antioxidants, and assesses health implications of degradation products. Key research gaps and future directions for improving frying practices and oil safety are also identified.

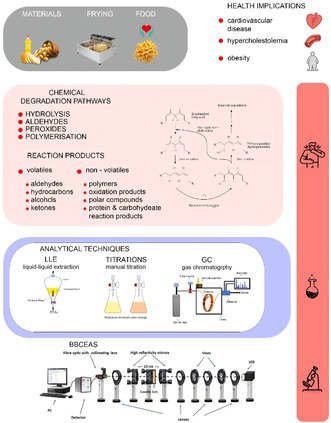

## 1 Introduction

### 1.1 Historical Perspective and Contemporary Practices

Frying is an ancient food processing method, with archaeological evidence indicating its use dating back thousands of years. Tomb decorations in ancient Egypt illustrate the use of frying, suggesting that both Europe and North Africa were familiar with the technique long before the modern era (Halawa 2023). Frying has been widely employed in both domestic and industrial sectors around the world (Zhang et al. 2020). The global demand for frying oils underscores the method's contemporary relevance, with an annual production estimated at around 20 million tonnes, which has since significantly increased (Sreelakshmi et al. 2022). Frying imparts unique sensory and organoleptic characteristics to food, enhancing its flavor, texture, and overall appeal (Negara et al. 2021). This cooking technique typically involves immersing food in edible oil at temperatures ranging from 150°C to 200°C, which promotes rapid cooking and the development of a desirable crispy texture (López et al. 2024a).

The growing popularity of frying is supported by the availability and economic viability of various vegetable oils such as sunflower, rapeseed, olive, and palm. Sunflower oil, extracted from *Helianthus annuus* , originated in North America and became commercially prominent in Eastern Europe (Aşkın and Kaya 2020). Ukraine, the world's leading exporter at 4.4 million tonnes annually, has seen its production disrupted by conflict, causing global price increases and rationing (Duke and Beaman 2022). Valued for its culinary use and health benefits, sunflower oil is rich in unsaturated fatty acids and Vitamin E, known to reduce cardiovascular risks and inflammation (Adeleke and Babalola 2020). Rapeseed oil, particularly the roasted variant, is favored for frying due to its neutral flavor and high smoke point. Once banned for high erucic acid content, modern low‐erucic varieties are now widely accepted, especially in the EU, where consumption is high (Zhang et al. 2024). It is also rich in omega‐3, omega‐6, and vitamins E and K. Olive oil, especially cold‐pressed “extra virgin” types, is rich in phenolic compounds with health‐protective properties (Majumder et al. 2022). Processed “pure” olive oil offers higher thermal stability for industrial use (Elshamy et al. 2021). Palm olein, with high oxidative stability and a healthier fatty acid profile, is common in EU snack production (Imoisi et al. 2015). Fundamental Principles of Deep‐Fat Frying.

Deep‐fat frying is a complex cooking process that involves several key principles to achieve the desired food quality. At its core, frying is the process of cooking food by immersing it in hot oil, typically at temperatures between 150°C and 200°C (Nayak et al. 2016). The high temperature facilitates rapid heat transfer, resulting in the quick cooking of the food's exterior while maintaining moisture and tenderness inside (Aguilera 2018).

A key physical mechanism is the transfer of heat from hot oil to the food, followed by internal conduction. This is accompanied by mass transfer, where water evaporates from the food and oil is absorbed, both of which influence texture and nutritional quality.

Chemically, one of the most critical reactions in deep‐fat frying is the thermal oxidation of unsaturated lipids. Unsaturated fatty acids present in frying oils are particularly susceptible to oxidation at high temperatures. This process leads to the formation of hydroperoxides and secondary oxidation products such as aldehydes, ketones, and alcohols, many of which contribute to rancidity and may pose health risks (Shahidi and Hossain 2022). The extent of oxidation depends on factors such as oil composition, frying duration, and exposure to oxygen.

Other significant reactions include the Maillard reaction, a non‐enzymatic browning process between amino acids and reducing sugars that enhances flavor and color (Chang et al. 2020), as well as starch gelatinization and protein denaturation, which contribute to the texture and structure of fried foods (Sivaranjani et al. 2024).

The choice of oil is crucial: oils with high smoke points and oxidative stability—such as sunflower, palm, and soybean oils—are preferred to minimize the formation of harmful degradation products (Gertz 2014). Furthermore, controlling frying parameters such as temperature and time is essential to regulate oil absorption and ensure the safety and sensory appeal of the final product (Zhang et al. 2020).

## 2 Chemical Mechanisms in Deep‐Fat Frying

### 2.1 Heat Transfer and Thermodynamics

Deep‐fat frying involves complex physical mechanisms, primarily heat and mass transfer, which collectively determine the kinetics of the process and the quality of the final product. Heat transfer in frying is highly efficient due to the high heat transfer coefficients of hot oil and the dynamic conditions within the fryer (Dehghannya and Ngadi 2021). Initially, heat is transferred from the hot oil to the surface of the cold food by convection. This is followed by conduction of heat from the surface into the interior of the food (Dash et al. 2020). As the food's internal temperature rises, the water content within the food begins to evaporate, generating vapor bubbles.

The rapid evaporation of water induces vapor turbulence around the food, which enhances heat transfer via forced convection, thereby accelerating the cooking rate (Lee and Taştemir 2023). However, if vapor bubbles become trapped and form a layer on the food surface, they can create an insulating barrier that hinders further heat transfer (Safari et al. 2018). Additionally, a portion of the input heat is consumed as latent heat for water evaporation, reducing the energy available for increasing the food's internal temperature.

In parallel with heat transfer, mass transfer plays a crucial role during frying. The most significant mass transfer processes include the migration of water from the food matrix to the surrounding oil and the simultaneous uptake of oil into the food (Miller et al. 1994). As water evaporates and escapes through the food's surface, a porous structure is formed, creating pathways for oil to infiltrate. This inward movement of oil is largely driven by capillary action and pressure differences created by vapor flux. The extent and rate of mass transfer are influenced by factors such as frying temperature, time, food composition, and the geometry of the food material.

Together, these heat and mass transfer mechanisms govern the kinetics of the frying process. They influence not only the rate of moisture loss and oil absorption but also the texture, crispiness, and moisture retention of the fried product (Huang et al. 2024). A comprehensive understanding of both mechanisms is therefore essential for optimizing frying conditions and ensuring product quality and safety.

### 2.2 Oil Degradation Processes

Edible oil degradation during frying is a complex process that involves various chemical reactions, including hydrolysis, oxidation, and polymerization. While these reactions contribute to oil deterioration and the formation of potentially harmful compounds with implications for human health and the environment, they also play a crucial role in developing the desirable sensory attributes of fried foods. Lipid oxidation is not only a degradation pathway but also essential in generating flavor compounds, aroma, and the characteristic golden‐brown color that enhance consumer appeal. Therefore, the chemical reactions occurring during deep‐fat frying should be evaluated from both a food quality and safety perspective.

#### 2.2.1 Hydrolysis

Hydrolysis is a critical degradation process in frying oils, primarily driven by the high moisture content in raw food materials (Asokapandian et al. 2020). When water interacts with oil at high temperatures, it breaks down triglycerides into free fatty acids, glycerol, and mono‐ and diglycerides (Menalla et al. 2024). This reaction reduces the oil's surface tension and can affect its oxidative stability. As frying continues, the concentration of free fatty acids (FFAs) in the oil increases, contributing to off‐flavors and making the oil less desirable for cooking (Shahidi and Hossain 2022). Hydrolysis mainly occurs within the oil phase rather than at the water–oil interface, with short and unsaturated fatty acids being more prone to hydrolysis due to their higher solubility in water. Factors such as frequent oil replacement and minimizing alkali contamination can slow down this process. The breakdown products from hydrolysis, being lower in molecular weight and higher in polarity, further accelerate the degradation of oil.

Hydrolysis mainly occurs within the oil phase rather than at the water–oil interface, with short and unsaturated fatty acids being more prone to hydrolysis due to their higher solubility in water. Factors such as frequent oil replacement and minimizing alkali contamination can slow down this process. The breakdown products from hydrolysis, being lower in molecular weight and higher in polarity, further accelerate oil degradation.

High levels of FFAs have been linked to inflammation and metabolic disorders. Regulatory limits for FFA content in frying oils vary by country. For example, in Germany, the maximum acceptable FFA content is set at 2.0%, while in France it is limited to 1.5%, and in the UK, a limit of 2.5% is applied for discarded oil (Welty et al. 2016; Fritsch 1981). These specific thresholds reflect efforts to protect consumer health while also maintaining the sensory quality of fried products.

#### 2.2.2 Oxidation

Oxidation is a primary degradation pathway in frying oils, occurring when oils are exposed to oxygen at high temperatures (Erickson et al. 2023). This reaction leads to the formation of hydroperoxides, which subsequently break down into secondary oxidation products such as aldehydes, ketones, and alcohols. Depending on the fatty acid profile of the frying oil, different profiles of oxidation volatiles can develop. For instance, high oleic peanuts have been shown to generate nonanal and 2‐undecenal as key oxidation volatiles. These compounds contribute to off‐flavors, rancidity, and potentially toxic effects. The formation of trans fats, which are associated with an increased risk of heart disease, is also a result of oxidation processes (López et al. 2024b). Studies have shown that these oxidation products can have deleterious effects on health, emphasizing the need for careful monitoring and control of frying conditions (Grootveld et al. 2021).

##### 2.2.2.1 Principle and Consequences

**FIGURE 1:**
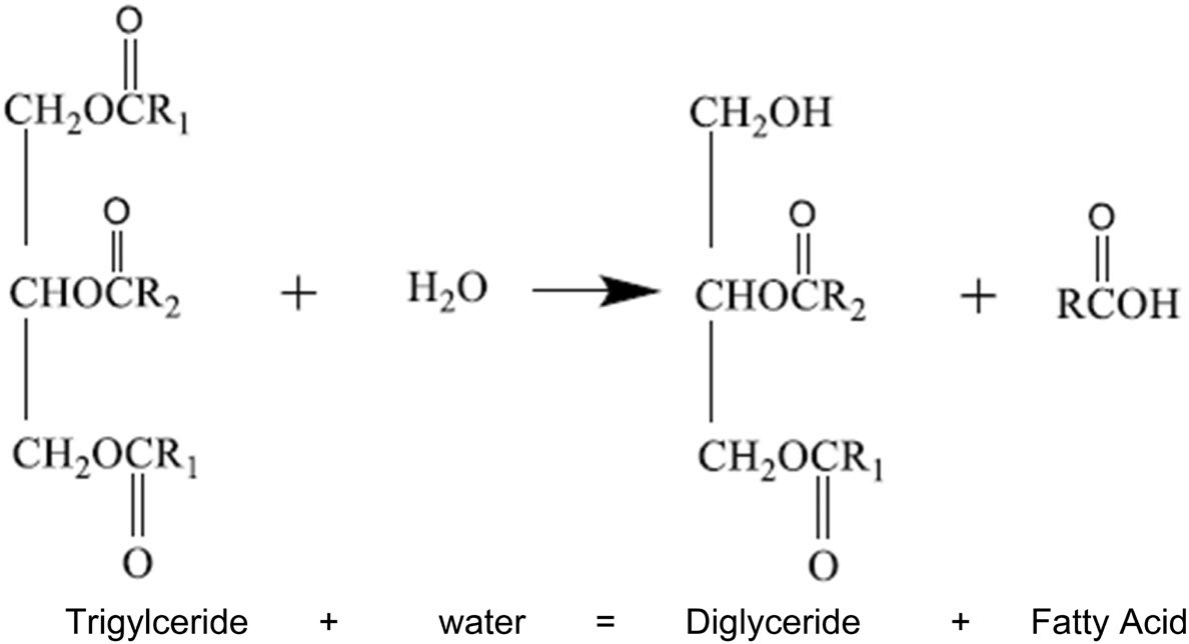
Hydrolytic reaction and products formed during frying (Welty et al. 2016).

**FIGURE 2:**
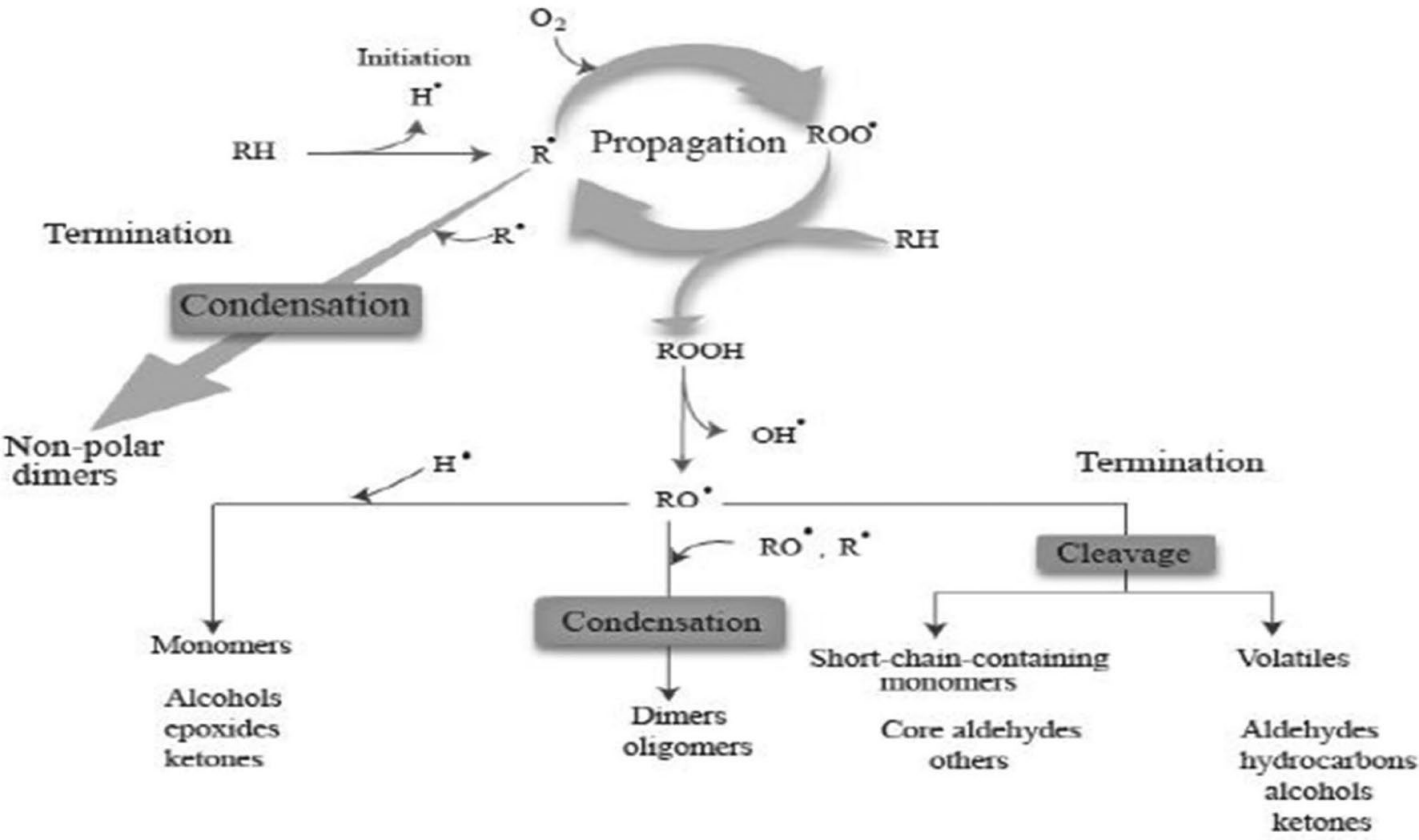
Mechanism of thermal oxidation (Frakolaki et al. 2023).

The complexity of lipid oxidation extends beyond the initial formation of hydroperoxides, encompassing a series of radical‐mediated chain reactions that ultimately lead to structural lipid alterations. These mechanisms are further elaborated in the subsequent discussion on autoxidation and thermal oxidation pathways (Rodriguez‐Amaya and Shahidi 2021). Apart from the reaction speed, the autoxidation process is largely the same as the thermal oxidation process occurring in frying (Shen et al. 2023). The mechanism of thermal oxidation is (Figure 1) summarized in Figure 2. The ester group is liable to hydrolytic changes, thus resulting in the decomposition of the oil molecules into glycerol and constituent fatty acids. Generally, all unsaturated and saturated fats undergo oxidative degradation, although through different mechanisms and at varying rates. While unsaturated fats oxidize readily due to their double bonds, saturated fats degrade more slowly, typically through hydrogen abstraction at the α‐position adjacent to the carboxyl group, forming alkyl radicals that initiate oxidation (Rodriguez‐Amaya and Shahidi 2021). Triglycerides are less susceptible to oxidative changes when compared to free fatty acids, and their presence in oil enhances the rate of oil rancidity (Machado et al. 2023).

In saturated fatty acids, the site of radical formation is different from that in unsaturated oleic and linoleic acids. For these compounds, the alkyl radical is formed at the α‐position of the carboxyl group (Mittu et al. 2023). The propagation step in lipid oxidation is prompted by the formation of peroxy radical intermediates and its subsequent reaction with fatty acids to form more alkyl radicals. The termination step consists of the irreversible breakdown of hydroperoxides and subsequently leads to the formation of various small molecules (Dodoo et al. 2022).

The rate of oxidation in oil decreases with the amount of antioxidants present. Deep frying leads to less oxidative reaction of oil compared to shallow frying, due to an increased oxygen interface in the later process (Yildiz et al. 2024). During frying at high temperatures, the oxidative stability of oil is affected by several other factors including FFA content, the presence of transition metals, light, oxygen, food type, moisture, the type of oil, food crumbs, salt and additives, and the frequency of oil usage (Bazina and He 2018; Bazina et al. 2022). The oxidation of oil may produce products such as ketones, acids, aldehydes, alcohols, and lactone (Rodriguez‐Amaya and Shahidi 2021). Monitoring the formation of these compounds using analytical techniques can provide an indication of oil quality indirectly.

##### 2.2.2.2 Primary and Secondary Oxidation

As illustrated in Figure 2, the oxidation process involves three stages: initiation, propagation, and termination (Shen et al. 2023). The initiation stage involves the removal of a hydrogen from a fatty acid chain to form an alkyl radical in the presence of oxygen. Various factors can shorten the reaction time of this stage, including heat, the presence of iron or copper, and UV light (with the presence of chlorophyll). Another fatty acid then reacts with a free radical to produce hydroperoxide with another alkyl free radical (propagation) (Kontogianni and Gerothanassis 2022). The reaction continues until there is a depletion of oxygen, a fatty radical reacts with a stable antioxidant radical, or unstable radicals react (termination) (Gulcin 2020).

The three‐step reaction mechanism involved in the thermal oxidation of oil is as outlined below:

- Initiation step—in unsaturated fatty acids, oxidation is initiated by a free radical reaction owing to factors including heat or light. The C‐H bond of the alpha methylene group is weakened by the strong electron affinity inherent between the neighboring carbon atoms of the unsaturated carbon–carbon double or triple bond. Homolytic hydrogen atom abstraction from a methylene group leads to alkyl radical (R*) formation;

(1) $$\mathrm{RH}\to R^{*}$$

b Propagation step—this is prompted by the formation of peroxy radical intermediates and its subsequent reaction with fatty acids to form more alkyl radicals and hydroperoxides:

(2) $$R^{*}+O_{2}⇌{\mathrm{ROO}}^{*}\;\left(\text{Fast}\right)$$

(3) $${\mathrm{ROO}}^{*}+\mathrm{RH}\to \text{ROOH}+R^{*}\;\left(\text{Slow}\right)$$

c Termination step—formation of non‐radical products by interaction of R* and ROO*;

(4) $$\begin{array}{c} R*+R*\to \mathrm{RR}\;\left(\text{non}-\text{radical product}\right)\\ R*+\mathrm{ROO}*\to \text{ROOR}\;\left(\text{non}-\text{radical product}\right)\\ \mathrm{ROO}*+\mathrm{ROO}*\to \text{ROOR}+O_{2}\;\left(\text{non}-\text{radical products}\right) \end{array}$$

where R* = fatty acids radical; ROOH = fatty acid hydroperoxide; ROO* = peroxide radical; R = lipid alkyl (Frakolaki et al. 2023).

#### 2.2.3 Polymerization

**FIGURE 3:**
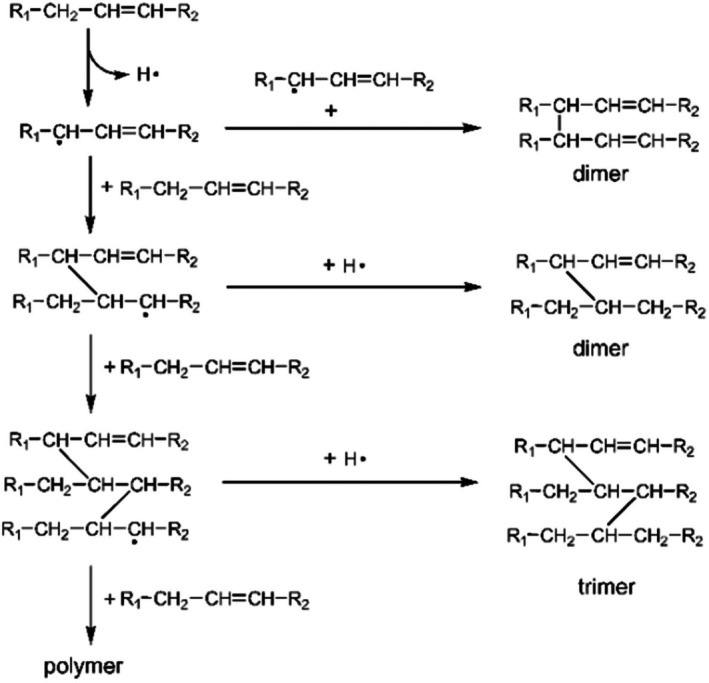
Production of acyclic polymer from oleic acid during deep‐fat frying (Frakolaki et al. 2023).

**FIGURE 4:**
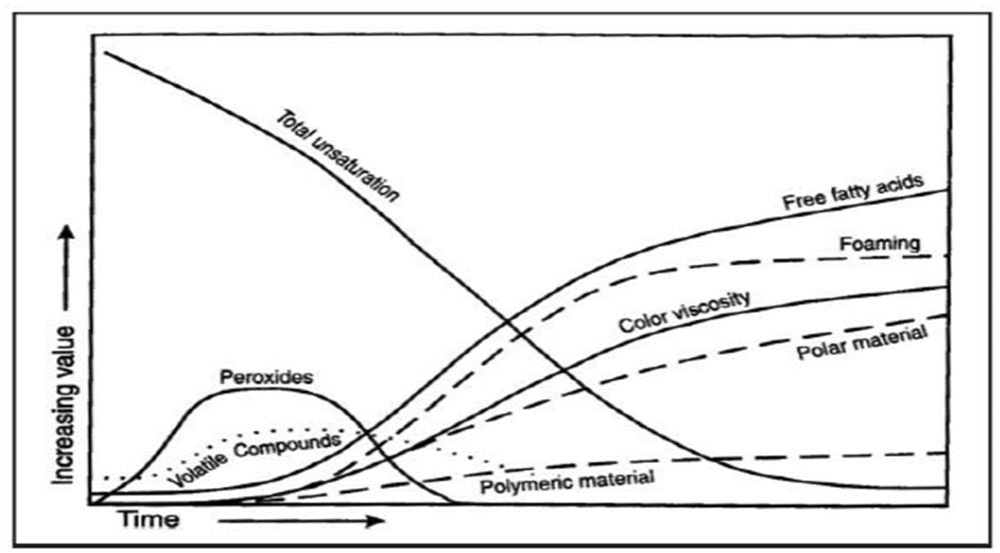
Physical and chemical changes in oil during deep‐fat frying (Frakolaki et al. 2023).

An important group of reactions involved in thermal oxidation is polymerization reactions as shown in Figure 3. During thermal oxidation, non‐volatile polar compounds, including cyclic monomers, conjugated dienes, triglyceride dimers, and polymers, are major decomposition products formed by polymerization (Chen et al. 2021). These polymers are formed by free radical chain reactions and are relatively high molecular weight compounds, with masses ranging between 692 and 1600 Da, composed of combinations of –C‐C–, –C‐O‐C–, and –C‐O‐O‐C bonds (Mittu et al. 2023). The concentration of dimers and polymers has been reported to be higher compared to products of cyclic compounds. For instance, dehydrodimer, monohydrodimer, ketodehydrodimer, and dehydroxydimer of linoleate can be detected in soybean oil at 195°C (Serna‐Saldivar 2022). Polymerization can produce cyclic or acyclic compounds, depending on the types of fatty acids present in the frying oil (Tripathi and Singh 2023). Oils rich in linoleic acid have been found to polymerize more than oils rich in oleic acid. The length and temperature of heating have a positive relationship with the rate of polymerization (Khor et al. 2019). The acceptable limit for dimers and polymers in frying oils is typically set between 10% and 16%, depending on the country and its specific regulations. This range is considered safe for consumption and ensures the quality of the frying process. For instance, a study published in the British Food Journal notes that frying oils with total polar materials (TPM) exceeding 25% are deemed non‐edible due to significant degradation, including the formation of polymers (Ahmadi et al. 2018). These reactions not only influence the physical and chemical properties of the oil but also raise health and environmental concerns. Polymerized oils, being more viscous, impede effective heat transfer, adversely affecting food quality. Furthermore, the difficulty in digesting these large molecules poses health risks (Wang et al. 2021). The high molecular weight of these compounds complicates oil filtration and recycling processes, thus contributing to environmental issues. The regulation of polymerization products is essential to mitigate these impacts and maintain the quality and safety of frying oils (Bhandari et al. 2020) (Figure 4).

## 3 Factors Influencing the Thermal Stability of Frying Oil

### 3.1 Temperature and Frying Time

Temperature and frying time are critical factors influencing the degradation of frying oil, significantly affecting its chemical stability and quality (Erickson et al. 2023). Elevated frying temperatures accelerate the rate of oxidation, polymerization, and thermal breakdown of oils. As the temperature increases, hydro‐peroxide decomposition and the formation of oxidation products are intensified. For instance, during potato chip frying, soybean oil at 170°C for 70 h resulted in 3.09% conjugated dienes and 1.68% trans‐fat, whereas at 190°C, these values increased to 4.39% and 2.60%, respectively (Ahmad et al. 2021). This demonstrates that higher temperatures not only promote the generation of undesirable compounds but also alter the chemical structure of the oil, increasing the number of polymers with ether or carbon‐to‐carbon linkages while reducing polymers with peroxide linkages.

Repeated heating and cooling cycles further exacerbate oil degradation, as cooling facilitates oxygen dissolution, thereby accelerating oxidation. The temperature also influences the decomposition of hydroperoxides, which occurs more rapidly at elevated temperatures. For example, crude herring oil shows faster hydroperoxide decomposition at 50°C in the dark compared to its formation. In contrast, at lower temperatures (20°C), the decomposition rate remains higher than the formation rate (Sørensen et al. 2023). Despite these effects, singlet oxygen (1O_2_) oxidation is minimally influenced by temperature due to its low activation energy requirement of 0 to 6 kcal/mol (Chilakamarthi et al. 2022).

Frying time further amplifies the degradation of oil. Prolonged usage leads to increased formation of free fatty acids (FFAs), polar compounds, dimers, and polymers. These degradation products accumulate proportionally with frying time and temperature, contributing to thermal oxidative degradation. However, some studies suggest that the accumulation of polar compounds eventually plateaus after extended frying durations (Shen et al. 2022). This indicates a potential limit to degradation under specific conditions, though the oil's quality remains significantly compromised. The combined effects of temperature and frying time underline the importance of optimizing frying practices to minimize oil degradation, preserve nutritional quality, and reduce the formation of harmful compounds (Valle et al. 2024).

### 3.2 Fatty Acid Composition

Triglycerides are the primary constituents of fats and oils, comprising three fatty acid units and one glycerol unit (Sharma et al. 2022). These compounds are soluble in organic solvents but insoluble in aqueous mediums. The triglyceride configuration varies depending on the source of fats and oils, influencing their physical and functional properties. Typically, vegetable oils contain medium‐chain‐length fatty acids (FAs) with even carbon atoms ranging from 12 to 24, with 18‐carbon FAs being the most prevalent (Frakolaki et al. 2023).

The fatty acid composition significantly affects the oxidative stability of oils during heating processes. The impact of fatty acids on thermal oxidative stability intensifies with prolonged heating. Studies indicate that the thermal oxidative stability of oils is influenced not only by fatty acid composition but also by the presence of antioxidants and other nutrients (Fadda et al. 2022). Generally, unsaturated fatty acids are more prone to oxidation compared to saturated fatty acids due to the presence of double bonds, which are highly reactive (Olmedo et al. 2018). The rate and quantity of primary oxidation products increase with the number of double bonds in fatty acids, particularly during prolonged frying periods. Furthermore, the reactivity of unsaturated fatty acids, including oxidation and polymerization, is heightened when double bonds are in conjugated positions (Biermann et al. 2021).

Table 1 illustrates the relative proportions of key saturated and unsaturated fatty acids in commonly used vegetable oils. This comparison highlights the variation in fatty acid profiles across oil types, which helps explain differences in their oxidative stability and suitability for high‐temperature frying applications. For instance, oils with higher oleic acid content (such as sunflower or olive oil) tend to be more stable than those with high linoleic acid content (such as corn oil), which are more prone to rapid oxidation.

**TABLE 1:** Fatty acid composition (%) of some vegetable oils (Awogbemi et al. 2019).

Fatty acid	Palm oil	Palm olein	Olive oil	Rapeseed oil	Sunflower oil	Corn oil
Palmitic acid C16:0	40–46	38–43	7.5–20	3.3–6	2.7–4.2	10–17
Steric acid C18:0	4–7	3.7–4.8	0.5–5.0	1.0–2.5	3.0–5.0	1.6–3.3
Oleic acid C18:1	36–41	40–44	55–83	52–67	80–87	25–42
Linoleic acid C18:2	9–12	10–13	3.5–21	16–25	4–9	39–61
Linolenic acid C18:3	0.1–0.4	0.1–0.6	0–1.5	6.0–14	—	0.7–1.3

### 3.3 Metals

Transition metals, such as iron and copper, significantly reduce the stability of edible oils by accelerating oxidative processes (Bazina et al. 2022). Acting as potent pro‐oxidants, these metals catalyze the breakdown of peroxides into highly reactive radicals, bypassing the initiation stage of oxidation and directly increasing the rate of oil degradation. The role of transition metals in this process can be explained through a two‐step mechanism shown in Equations (5) and (6). In the first step, Fe^2+^ or Cu^+^ cleaves the bond between the oxygen atoms in alkyl hydroperoxides, resulting in the formation of an alkoxyl radical (RO*) and a hydroxide ion (OH^−^):

(5) $$\text{ROOH}+{\mathrm{Fe}}^{2+}\;\left(\text{or}\;{\mathrm{Cu}}^{+}\right)\to {\mathrm{RO}}^{*}+{\mathrm{Fe}}^{3+}\;\left(\text{or}\;{\mathrm{Cu}}^{2+}\right)+{\mathrm{OH}}^{-}$$

In the second step, Fe^3+^ or Cu^2+^ further cleaves the bond between hydrogen and oxygen in the alkyl hydroperoxide, producing a peroxyl radical (ROO*) and regenerating Fe^2+^ or Cu^+^:

(6) $$\text{ROOH}+{\mathrm{Fe}}^{3+}\;\left(\text{or}\;{\mathrm{Cu}}^{2+}\right)\to {\mathrm{ROO}}^{*}+{\mathrm{Fe}}^{2+}\;\left(\text{or}\;{\mathrm{Cu}}^{+}\right)+{\mathrm{OH}}^{-}$$

Through this catalytic process, transition metals not only accelerate the decomposition of hydroperoxides but also generate secondary oxidation products such as aldehydes, ketones, and short‐chain hydrocarbons, which further degrade the oil's sensory qualities (Rodriguez‐Amaya and Shahidi 2021). These compounds negatively affect the sensory qualities of oils, altering their taste, odor, and flavor (Mahanta et al. 2021).

Even trace levels of iron and copper can drastically reduce the oxidative stability of edible oils. Their presence accelerates the formation of undesirable compounds, significantly impacting the oil's freshness and quality. The catalytic activity of these metals in the decomposition of hydroperoxides highlights their detrimental role in the autoxidation process (Machado et al. 2023). Furthermore, the susceptibility of oils to oxidation increases proportionally with the concentration of these metals, making their control and monitoring critical for maintaining oil quality during processing and storage (Flores, Meyer, et al. 2021). The oxidative instability caused by metals underscores the importance of refining processes, which effectively reduce metal concentrations and improve oil stability. Understanding the role of transition metals in oil oxidation is essential for developing strategies to mitigate their impact, whether through refining, storage conditions, or innovative analytical methods for detecting trace metal levels. These efforts are vital for ensuring the longevity and sensory integrity of edible oils (Guharoy et al. 2021).

### 3.4 Fryer Design and Surface‐To‐Volume Ratio

The design of a fryer significantly influences the rate at which frying oil deteriorates (Erickson et al. 2023). One critical factor is the surface‐to‐volume ratio of the oil, which affects the extent of oil exposure to oxygen and heat. An increase in the surface‐to‐volume ratio leads to a greater oil surface area exposed to air, thereby enhancing the magnitude of oxidation (Machado et al. 2023). This results in accelerated oil degradation due to increased rates of oxidative reactions. To mitigate this effect, fryer designs can be optimized by modifying the oil depth‐to‐area ratio, defined as ($R=\frac{D}{A^{1/2}}$), where D represents the depth of the oil and A denotes the surface area of the oil. A higher value of R reduces the surface area exposed to air per unit volume of oil, thereby slowing down oxidation and improving oil stability during frying. This design principle highlights the importance of balancing fryer dimensions to reduce oxidative stress on the oil while maintaining frying efficiency (Majchrzak et al. 2021). Fryers with deep oil levels and smaller surface areas minimize the contact between oil and oxygen, thus extending the oil's usable life (Su et al. 2024). Additionally, optimizing the fryer's design can reduce the thermal load on the oil, as a smaller exposed surface reduces heat dissipation and stabilizes the oil's temperature.

### 3.5 Moisture Content

During deep‐fat frying, moisture is continuously transferred from food into the oil, which significantly influences the hydrolytic degradation of the oil. Increased moisture content in foods accelerates oil hydrolysis under frying conditions, leading to decreased oxidative stability (Yildiz et al. 2024). The moisture content of oils used in frying processes, such as those for chips and breaded chicken, plays a critical role in determining their oxidative stability (Shen et al. 2023).

The frying process inherently causes moisture loss from food, as elevated temperatures commonly exceeding 150°C promote drying (Yildiz et al. 2024). Higher frying temperatures result in a more rapid reduction of moisture content. Managing moisture levels in frying oils is essential for minimizing lipid degradation and maintaining oil quality. Interestingly, small amounts of moisture (approximately 0.7%) have been shown to exert a protective effect by forming a thin film on the oil's surface, which can help reduce oxidation (Dana et al. 2003). However, excessive moisture levels can catalyze undesirable reactions, leading to the development of off‐flavors and compromising oil stability.

Degraded or rancid oils often exhibit reduced surface tension, a phenomenon linked to the chemical changes occurring during degradation. The decomposition of hydroperoxides generates amphiphilic compounds, which contain both hydrophilic and hydrophobic properties (Budilarto and Kamal‐Eldin 2015). These compounds not only lower surface tension but also facilitate oxygen introduction into the oil, thereby accelerating oxidative deterioration. Effective control of moisture during frying is, therefore, a critical factor in preserving the thermal stability and quality of frying oils (Aladedunye 2015).

### 3.6 Oil Production Method

The production methods employed in the processing of oils play a critical role in determining their oxidative stability (Abeyrathne et al. 2021). Commercial fats and oils are obtained through extraction and refining processes, which can introduce variability in the oxidative stability of the final products (Redondo‐Cuevas et al. 2018). These processes often involve exposure to high temperatures, contact with metals, and interaction with light, oxygen, and moisture, all of which can accelerate oxidative degradation (Machado et al. 2023). Refining processes, as detailed in Table 2, can alter the minor components in oils that are crucial for maintaining oxidative stability.

**TABLE 2:** Effect of processing steps on the removal of minor components in oils processing (Gharby 2022).

Process step	Minor components removed
Degumming	Phospholipids
Refining	Free fatty acids, phospholipids, metal ions and soaps
Bleaching	Pigments, primary oxidation products
Fractionation	Waxes, solid triacylglycerols
Deodorization	Free fatty acids, secondary oxidation products, residual pigments, sterols, hydrocarbons, other volatiles

Components such as tocopherols and other unsaponifiable substances, which are naturally present in crude oils, contribute significantly to their high oxidative resistance (Ayyildiz et al. 2015). However, these beneficial compounds are often reduced or removed during refining, making refined oils more susceptible to oxidation compared to their crude counterparts. Furthermore, oxidative changes may occur during the storage and packaging of bulk oils, especially under suboptimal conditions (El Guerraf et al. 2024). The method of extraction also impacts the oxidative stability of oils (Olmedo et al. 2018). For instance, walnut oil obtained through supercritical fluid extraction has been reported to exhibit lower oxidative stability compared to pressed oil (Gao et al. 2022). This difference highlights the importance of production techniques in preserving the chemical integrity and quality of oils. In addition to the impact of extraction methods and the loss of natural antioxidants during refining, specific components removed or retained through processing also play a crucial role in the oxidative stability of oils. The elimination of free fatty acids (FFAs) during refining is particularly important, as FFAs are more prone to oxidation than when they are esterified within triglyceride structures. Their presence can accelerate oxidative degradation, reducing the shelf‐life and functional quality of the oil. Moreover, phospholipids commonly removed during degumming contribute to increased foaming during deep frying (Islam et al. 2023). This foaming expands the oil's surface area in contact with oxygen, thereby promoting oxidation (Zhou et al. 2024). To mitigate these effects, industrial frying processes often incorporate defoamers, which help control foam formation and reduce oxidative stress on the oil during high‐temperature use (Mounts 2018).

### 3.7 Light and Oxygen

UV light or sunlight accelerates oil oxidation in the presence of photosensitisers such as chlorophyll in a process known as singlet oxygen oxidation (Lopes and Courrol 2023). Light absorption by photosensitisers occurs very rapidly, in picoseconds, causing the photosensitisers to become excited. These excited photosensitisers can initiate lipid oxidation themselves or react with triplet oxygen to produce singlet oxygen, which reacts with unsaturated fatty acids 1500 times faster than triplet oxygen (Wang, Xiao, et al. 2023). Oil oxidation is also significantly influenced by the type of oxygen and its concentration. For instance, when high‐oleic acid safflower oil is exposed to an atmosphere containing 2% oxygen, volatile carbonyl compounds, particularly acetaldehydes, are formed (Fujisaki et al. 2002). In a 20% oxygen environment, compounds such as hexanal and nonanal become most prevalent. The reaction between edible oil and oxygen becomes more efficient when the oil sample has a high surface area‐to‐volume ratio or when the sample size is small (Gigi et al. 2024). Volatile compound formation in rapeseed oil is influenced by the interaction between temperature and oxygen concentration, even in the dark. As the oxygen concentration in the oil increases, the amount of volatile aldehydes emitted during the thermal treatment of high‐oleic safflower oil also rises (Fujisaki et al. 2002). Furthermore, the flavor of fried products is affected by the intensity of the oxygen concentration. The degree of interface between the air and the frying oil is facilitated by the presence of surface‐active compounds. Oil oxidation is further accelerated by free fatty acids, which reduce the oil's surface tension, thereby increasing the rate of oxygen dispersion (Berton‐Carabin et al. 2014). As highlighted above, oil oxidation mediated by singlet oxygen occurs at a much higher reaction rate compared to that of triplet oxygen.

Additionally, the use of defoamers in industrial frying processes can help mitigate oxidation by reducing foam formation, which otherwise increases the oil's surface area in contact with oxygen. By limiting this interface, defoamers contribute to reducing oil deterioration caused by oxygen exposure during high‐temperature applications (Deotale et al. 2023; Singh and Kishore 2023).

### 3.8 Phospholipids, Natural, and Synthetic Antioxidants

Phospholipids such as phosphatidylinositol, phosphatidic acid, phosphatidylserine, and phosphatidylcholine are key components of oils, playing a dual role in oxidative stability (Abdullah 2024). At low concentrations, typically below 60 ppm, these compounds act as antioxidants by stabilizing free radicals and chelating metal ions, which enhances the shelf life and thermal stability of oils (Cui and Decker 2016). However, at higher concentrations, phospholipids can transition to pro‐oxidant behavior, facilitating oxidative reactions by interacting with reactive oxygen species or promoting the breakdown of hydroperoxides into free radicals. This dual nature necessitates careful management of phospholipid levels during processing and storage to optimize oil stability, particularly in high‐temperature applications like frying, where oxidative stress is intensified (Costa et al. 2024).

Antioxidants, both natural and synthetic, are widely used to inhibit oil oxidation (Bazina et al. 2025). Naturally occurring antioxidants in oils include tocopherols, tocotrienols, sterols, carotenoids, and phenols, which mitigate oxidation through mechanisms like metal sequestration, singlet oxygen quenching, and free radical scavenging (Choe et al. 2019). Synthetic antioxidants such as butylated hydroxyanisole (BHA), butylated hydroxytoluene (BHT), propyl gallate (PG), and tert‐butylhydroquinone (TBHQ) are effective under specific conditions. However, their efficiency often decreases at frying temperatures due to volatilization or decomposition, with compounds like TBHQ showing better thermal stability than others. Despite this, significant losses of TBHQ and other antioxidants occur during frying, attributed to factors like thermal decomposition, steam distillation, and absorption by fried foods, highlighting the challenges in maintaining antioxidant effectiveness in such processes (Oyom et al. 2024). In light of these limitations, attention has increasingly turned to natural alternatives such as terpenes, which possess phenolic or non‐phenolic structures and have demonstrated protective effects on both frying oils and fried foods during the frying process. Moreover, certain terpenes may continue to exert antioxidant effects during subsequent storage, potentially improving the overall oxidative stability of the final product (Juncos et al. 2025).

### 3.9 Oil Replenishment

The practice of adding fresh oil during the deep‐frying process has been shown to significantly extend the frying life of the oil by reducing thermoxidative and hydrolytic changes (Valle et al. 2024). Regular replenishment slows the hydrolysis of oils such as sunflower oil, thereby lowering the production of undesirable compounds, including polar materials and free fatty acids (FFAs) (Sharma et al. 2022). In industrial frying, replenishment serves a dual purpose: maintaining the oil level in the fryer and preserving the quality of the frying oil. Studies indicate that better oil quality is achieved when the ratio of fresh oil to the total oil in the fryer is high, as this dilutes the concentration of degradation products (Sayyad 2017). By reducing the buildup of harmful by‐products, fresh oil addition enhances both the physical and chemical properties of the frying oil, such as its oxidative stability, viscosity, and color.

Furthermore, “the frequency and amount of replenishment are crucial factors influencing the frying process's efficiency and product quality” (Hosseini et al. 2016). Consistent addition of fresh oil helps maintain desirable sensory attributes in fried products, prolongs the usability of the oil, and reduces operational costs by delaying the need for complete oil replacement (Cui et al. 2024). This practice is particularly critical in large‐scale industrial frying operations, where the economic and quality benefits of controlled oil replenishment are substantial.

### 3.10 Food Material Composition

The thermal oxidation of oil in the presence of food proceeds at a slower rate compared to heating oil without any food (Flores, Meyer, et al. 2021). However, the type, size, and composition of food can significantly impact the stability of oil. Factors such as moisture and starch content contribute to oil degradation, with starch accelerating the process, while amino acids have a protective effect against degradation during deep‐fat frying (Flores, Avendaño, et al. 2021). Additionally, the presence of transition metals in food, such as iron found in meat, can increase oxidation rates. To mitigate such effects, the application of edible films, like hydroxyl propyl methylcellulose, has been shown to decrease the degradation of frying oil.

The interaction between amino acids and reducing sugars, through the Maillard reaction, also influences oil degradation (Guo et al. 2022). The rate of these reactions is dependent on variables such as water activity, chemical composition of the food, pH, and reaction temperature. During high‐temperature frying, both physical and chemical changes occur, which can greatly affect the thermal oxidative stability of the oil. However, these changes can also lead to the formation of harmful compounds, including potential carcinogens, posing risks to human health (Erickson et al. 2023). The chemical and physical changes in food during the frying process are summarized in Table 3.

**TABLE 3:** Main changes in the composition of foods during the frying process (Rani et al. 2023).

Component	Change during frying
Fat	Increased concentration, and change in composition
Water	Significant loss
Reducing sugars	Maillard reaction
Starch	Gelatinization
Proteins	Alteration of the composition
Amino acid	Formation of heterocyclic flavoring substances

## 4 Techniques

### 4.1 Conventional Methods for Assessing Frying Oil Stability

Conventional methods for evaluating frying oil stability rely on chemical and physical analyses, including free fatty acid (FFA) content, peroxide value (PV), anisidine value (AnV), total polar material (TPM), iodine value (IV), viscosity, color, and smoking point as shown in Table 4 (Vishwakarma et al. 2023; Frakolaki et al. 2023). Among these, titration is the most commonly used method for assessing FFA content due to its simplicity and widespread regulatory approval. It involves neutralizing the acids in oil with a strong base, but it has several limitations (Mahesar et al. 2014). The method requires large sample sizes (20–50 g), extensive use of solvents, and accurate endpoint detection, which can be challenging, especially for highly colored crude oils. Additionally, titration does not provide information on the specific FFA profile, limiting its ability to identify whether the acids formed due to hydrolysis or oxidation.

**TABLE 4:** Conventional and analytical methods for assessing frying oil stability.

Criteria	Conventional methods	Analytical methods
Examples	Titration (FFA), PV, AnV, TOTOX, INTOX, TPM, viscosity, color, smoke point	GC, LC, HPLC, MS, UV–Vis, IR, BBCEAS
Measured parameters	FFA content, primary and secondary oxidation products, polar compounds, physical attributes	Specific FFA profiles, aldehydes, antioxidants, trace metals, degradation products
Advantages	Simple, low cost, widely accepted by regulators, suitable for field/lab use	High sensitivity and specificity, reduced sample requirements, quantitative and qualitative data, improved detection limits
Limitations	Larger sample size, solvent use, low specificity, limited sensitivity, endpoint detection difficulties	Higher cost, requires equipment and trained personnel, less accessible in low‐resource settings
References	Vishwakarma et al. 2023; Frakolaki et al. 2023; Mahesar et al. 2014; Morales and Przybylski 2013; Karami et al. 2020; Juncos et al. 2024; Bansal et al. 2010	Bazina and He 2018; Bazina et al. 2022; Bazina et al. 2025

Similarly, peroxide value (PV) tests measure primary oxidation products in oil but are often unreliable for frying oils, as peroxides degrade rapidly into secondary oxidation compounds (Morales and Przybylski 2013). Anisidine value (AnV) assesses aldehyde formation, particularly secondary oxidation products, which contribute to undesirable odors in oil (Karami et al. 2020). Given the limitations of relying solely on PV or AnV, more integrative indicators such as the Total Oxidation Value (TOTOX) have been proposed to provide a more comprehensive measure of oil oxidation. The TOTOX value combines both PV and AnV (TOTOX = 2PV + AnV), offering a broader overview of the oxidative state of the oil by accounting for both primary and secondary oxidation products. Furthermore, in advanced stages of lipid degradation, the Integral Oxidation Value (INTOX) has been suggested as a more holistic metric, encompassing multiple oxidative changes throughout the frying process. These indices may serve as more robust tools for monitoring oil quality, especially under high‐temperature conditions where dynamic oxidation reactions occur (Juncos et al. 2024). Total polar material (TPM) measurement helps determine the extent of oil degradation and is regulated in many countries, though its assessment requires specific extraction procedures. Physical tests such as viscosity, color, and smoking point changes provide indirect quality assessments but lack precision and specificity in identifying the underlying degradation mechanisms (Bansal et al. 2010).

### 4.2 Analytical Methods for Assessing Frying Oil Quality

Instrumental analytical methods provide more precise and detailed assessments of frying oil quality compared to conventional techniques (see Table 4). These include Gas Chromatography (GC), Liquid Chromatography (LC), High‐Performance Liquid Chromatography (HPLC), Mass Spectrometry (MS), Ultra‐Violet Visible (UV–Vis) Spectroscopy, and Infra‐Red (IR) Spectroscopy. These methods offer quantitative and qualitative evaluations of oil degradation, oxidation, and contamination, ensuring more reliable monitoring of chemical changes during frying. In the authors' previous works, advanced techniques were explored to improve oil quality assessment. One study developed a method for identifying and quantifying free fatty acids (FFAs) in frying oil, using liquid–liquid extraction, esterification with boron trifluoride (BF_3_), and Gas Chromatography (GC) analysis. Requiring only 100 mg of oil, this approach demonstrated high efficiency in separating FFAs and revealed discrepancies compared to conventional titration, indicating that traditional methods may underestimate actual FFA content (Bazina and He 2018). Another study applied Broadband Cavity Enhanced Absorption Spectroscopy (BBCEAS) to detect trace metals, iron and copper, in edible oils, as these metals accelerate oxidation and oil degradation. This technique significantly improved sensitivity, detecting iron and copper at parts per billion (ppb) levels, highlighting its potential for oil quality monitoring (Bazina et al. 2022). Further research integrated HPLC with BBCEAS to enhance antioxidant detection, particularly BHA, BHT, and TBHQ in deep‐UV wavelengths (< 300 nm). Compared to conventional HPLC, this method lowered detection limits up to 30 times, offering a more cost‐effective and accurate way to assess antioxidant stability in frying oils (Bazina et al. 2025). These instrumental methods overcome the limitations of conventional techniques, providing greater sensitivity, specificity, and reduced sample requirements. Their application enhances the monitoring of frying oil degradation, ensuring better food safety, extended shelf life, and improved quality control in the fried food industry.

## 5 Types of Reaction Products in Deep‐Fat Frying

During the deep‐fat frying process, both volatile and non‐volatile compounds are generated as a result of thermal decomposition, oxidation, and interactions between food components and the frying medium (Chang et al., 2020). Volatile compounds primarily contribute to the aroma and flavor of fried foods, while non‐volatile compounds can significantly impact the safety and quality of the food.

### 5.1 Volatile Compounds: Identification and Impact

Volatile compounds are primarily responsible for the characteristic aroma and flavor of fried foods. These compounds are formed through the Maillard reaction, lipid oxidation, and thermal degradation of food components (Chang et al. 2020). Common volatile compounds include aldehydes, ketones, alcohols, and hydrocarbons. The presence and concentration of these compounds are influenced by factors such as frying temperature, oil type, and food composition (Atamaleki et al. 2022). While many volatile compounds enhance the sensory attributes of fried foods, some can be undesirable, imparting off‐flavors or contributing to the formation of potentially harmful substances like acrylamide (Li et al. 2023).

### 5.2 Non‐Volatile Compounds: Analysis and Implications

Non‐volatile compounds formed during deep‐fat frying include polymers, cyclic compounds, free fatty acids, and glycerides (Nanayakkara et al. 2020). These compounds result from the hydrolysis, oxidation, and polymerization of frying oils and food lipids. Non‐volatile compounds can degrade oil quality by increasing viscosity, reducing smoke point, and contributing to off‐flavors (Drabińska et al. 2023). Moreover, the accumulation of these compounds can pose health risks, as some are associated with toxicological concerns. For instance, the formation of trans fats and oxidized lipids during prolonged frying has been linked to cardiovascular diseases and other health issues. Therefore, regular monitoring and management of non‐volatile compounds in frying oil are essential for maintaining food safety and quality (Manzoor et al. 2022).

### 5.3 Impact of Reaction Products on Flavor and Aroma

The sensory attributes of fried foods, particularly their flavor and aroma, are heavily influenced by the reaction products formed during frying. Volatile compounds such as aldehydes, ketones, and alcohols are crucial in defining the desirable characteristics of fried foods (Panigrahi et al. 2023). These compounds result from the Maillard reaction between amino acids and reducing sugars, as well as the thermal degradation of fats. However, not all reaction products are beneficial. Off‐flavors can develop from the oxidation of unsaturated fats, leading to rancid notes that detract from the overall sensory experience. Furthermore, the balance and concentration of these compounds are affected by frying conditions such as temperature, duration, and the type of oil used (Chang et al. 2020). Understanding the formation and impact of these reaction products is essential for optimizing the sensory quality of fried foods while minimizing the production of harmful compounds (Wang, McClements, et al. 2023).

## 6 Health Implications of Deep‐Fat Frying

Excessive consumption of deep‐fat fried foods poses significant health risks due to the formation of harmful compounds during the frying process. Prolonged and repeated use of frying oils leads to the production of trans fats, which contribute to atherosclerosis, and the degradation of essential fatty acids. Toxic by‐products such as polar compounds, aldehydes, and heterocyclic amines (HAAs) have been linked to diseases like cancer, Parkinson's, Alzheimer's, hypertension, and metabolic disruptions. Additionally, the Maillard reaction, lipid oxidation, and carbohydrate degradation during frying generate harmful substances like furan and acrolein. While air‐frying methods reduce these toxic compounds, they may compromise sensory satisfaction compared to traditional deep‐fat frying. A summary of the major health conditions associated with deep‐fat frying, their underlying mechanisms, and possible mitigation strategies is provided in Table 5.

**TABLE 5:** Health implications of deep‐fat frying.

Health condition	Primary harmful compounds	Pathophysiological effects	Mitigation strategies	References
Heart disease	Trans fats, polar compounds, aldehydes	Raises LDL, lowers HDL; promotes systemic inflammation, oxidative stress, and endothelial dysfunction; increases risk of myocardial infarction, stroke, and arterial stiffness	Air frying, reducing fried food consumption, awareness campaigns	Shao et al. 2020; Dangal et al. 2024; Leong 2021; Qin et al. 2021; Flores, Meyer, et al. 2021; de Oliveira et al. 2024; Nestel et al. 2021
Hypertension	Trans fats, aldehydes, polar compounds, sodium	Vascular dysfunction, systemic inflammation, obesity‐related pressure load, and fluid retention	Low‐sodium options, steaming, air frying, dietary fat quality improvement	Fuchs and Whelton 2020; Dangal et al. 2024; Leong 2021; Clayton et al. 2023
Obesity	Calorie‐dense fats, trans fats, refined carbohydrates	Insulin resistance, increased visceral fat deposition, disrupted lipid metabolism, reduced satiety, overeating	Caloric moderation, high‐fiber alternatives, non‐frying methods	Grundy 2004; Dangal et al. 2024; Doucet and Tremblay 1997; Grootveld et al. 2020; Micha and Mozaffarian 2009; Kwon 2016
Hypercholesterolaemia	Trans fats, oxidized lipids, saturated fats	Increases LDL, lowers HDL; promotes oxidized cholesterol accumulation; leads to plaque formation and inflammation	Baking, steaming, replacing trans/saturated fats with healthier oils	van Rooy and Pretorius 2014; Reiner 2017; Dangal et al. 2024; Kontush 2024; Severino et al. 2020; Gadiraju et al. 2015; Oyom et al. 2024

### 6.1 Heart Disease

Heart disease, particularly coronary artery disease (CAD), is a leading global cause of death and is strongly influenced by dietary habits (Shao et al. 2020). Consumption of deep‐fat fried foods significantly increases the risk of heart disease due to harmful compounds generated during frying (Dangal et al. 2024). Trans fats, formed in prolonged frying, disrupt lipid profiles by raising LDL cholesterol and lowering HDL cholesterol, key contributors to atherosclerosis. Additionally, frying oils degrade into toxic polar compounds and aldehydes, which promote systemic inflammation, oxidative stress, and endothelial dysfunction, all precursors to cardiovascular disease (CVD) (Leong 2021).

Epidemiological studies link frequent consumption of fried foods to heightened risks of myocardial infarction and stroke (Qin et al. 2021). Regular intake is also associated with arterial stiffness and hypertension, further exacerbating cardiovascular risk. For example, individuals eating fried foods over four times a week face a significantly higher likelihood of heart disease compared to less frequent consumers (Flores, Avendaño, et al. 2021). Air frying has emerged as a healthier alternative, reducing the formation of trans fats and toxic by‐products (de Oliveira et al. 2024). However, sensory differences may hinder its adoption, particularly in regions where deep‐fat frying is culturally ingrained. To reduce the global burden of heart disease, public health measures should encourage healthier cooking methods, reduce fried food consumption, and promote awareness of its cardiovascular risks (Nestel et al. 2021).

### 6.2 Hypertension

Hypertension, or high blood pressure, is a major risk factor for cardiovascular diseases and is significantly influenced by diet (Fuchs and Whelton 2020). The frequent consumption of deep‐fat fried foods has been associated with an increased risk of hypertension due to the harmful compounds produced during the frying process (Dangal et al. 2024). These compounds, including trans fats, aldehydes, and polar compounds, contribute to vascular dysfunction and systemic inflammation, both of which play key roles in the development of high blood pressure (Leong 2021). Fried foods are also calorie‐dense and often lead to weight gain and obesity, which are significant contributors to hypertension. Excess body weight increases the workload on the cardiovascular system, leading to elevated blood pressure (Clayton et al. 2023). Furthermore, the high sodium content of many fried foods exacerbates fluid retention and vascular resistance, further increasing hypertension risk.

Epidemiological studies consistently show a positive correlation between fried food consumption and hypertension. For instance, individuals with diets rich in fried foods are more likely to develop high blood pressure than those who consume fried foods sparingly. Reducing the intake of fried foods and adopting healthier cooking alternatives, such as air frying or steaming, may lower hypertension risk. Public health initiatives promoting awareness of these risks and encouraging dietary changes are critical to managing hypertension globally.

### 6.3 Obesity

Obesity is a global health crisis linked to numerous metabolic disorders, including cardiovascular disease, type 2 diabetes, and hypertension (Grundy 2004). The excessive consumption of deep‐fat fried foods is a significant contributor to obesity due to their high caloric density and poor nutritional quality (Dangal et al. 2024). Deep‐fat frying increases the fat content of foods, making them energy‐dense and more likely to contribute to excessive calorie intake. The consumption of such foods often leads to weight gain due to an imbalance between calorie intake and energy expenditure (Doucet and Tremblay 1997). Additionally, fried foods are commonly prepared with refined carbohydrates and unhealthy fats, which have been shown to promote insulin resistance and fat accumulation.

The harmful compounds formed during frying, such as trans fats and oxidized lipids, may further exacerbate obesity by disrupting normal metabolic processes (Grootveld et al. 2020). Studies suggest that trans fats interfere with lipid metabolism, leading to increased visceral fat deposition, a key risk factor for metabolic syndrome (Micha and Mozaffarian 2009; Kwon 2016). Furthermore, frequent consumption of fried foods is associated with reduced satiety and increased cravings, leading to overeating and poor dietary habits. Public health measures should focus on reducing fried food consumption and promoting healthier cooking methods such as air frying, grilling, or steaming. Raising awareness of the link between fried foods and obesity is essential for encouraging healthier dietary choices and combating obesity‐related diseases.

#### 6.3.1 Hypercholesterolemia

Hypercholesterolaemia, characterized by elevated blood cholesterol levels, is a major risk factor for cardiovascular diseases, including atherosclerosis and coronary artery disease (van Rooy and Pretorius 2014; Reiner 2017). Dietary habits play a crucial role in cholesterol metabolism, and the frequent consumption of deep‐fat fried foods is strongly associated with an increased risk of hypercholesterolaemia (Dangal et al. 2024). During deep‐fat frying, oils undergo chemical degradation, leading to the formation of trans fatty acids and oxidized lipids. Trans fats are particularly harmful as they increase low‐density lipoprotein (LDL) cholesterol, commonly referred to as “bad” cholesterol, while simultaneously decreasing high‐density lipoprotein (HDL) cholesterol, which has protective cardiovascular effects (Kontush 2024). This imbalance contributes to arterial plaque formation, increasing the risk of heart disease and stroke (Severino et al. 2020).

Fried foods are also high in saturated fats, which further elevate LDL cholesterol levels. Additionally, repeated heating of frying oils results in the production of oxidized cholesterol, which has been linked to endothelial dysfunction and inflammation, accelerating the progression of cardiovascular disease. Epidemiological studies indicate a strong correlation between frequent fried food consumption and hypercholesterolaemia (Gadiraju et al. 2015). Reducing the intake of deep‐fried foods and opting for healthier cooking alternatives, such as air frying, baking, or steaming, may help regulate cholesterol levels and lower cardiovascular risks (Oyom et al. 2024). Public health initiatives should focus on raising awareness about the impact of dietary choices on cholesterol metabolism.

## 7 Discussion

Understanding the chemical and physical transformations that occur during deep‐fat frying is essential for ensuring both product quality and consumer health. The degradation of oils through oxidation, hydrolysis, and polymerization represents a complex network of interrelated reactions, each influenced by a range of intrinsic and extrinsic factors. Rather than occurring in isolation, these reactions form a dynamic cascade, where the products of one pathway often serve as reactants or catalysts in another. For example, hydrolysis generates free fatty acids (FFAs), which in turn lowers the surface tension of the oil and increases its susceptibility to oxidation. Similarly, oxidation products such as aldehydes and ketones not only affect sensory quality but can also promote polymerization under prolonged thermal exposure.

Fatty acid composition remains a central determinant of oil behavior under frying conditions. Oils rich in monounsaturated fatty acids, such as oleic acid, exhibit greater oxidative stability compared to those dominated by polyunsaturated fatty acids, such as linoleic and linolenic acids. However, it is not only the degree of unsaturation but also the positional arrangement of double bonds—particularly the presence of conjugated systems—that can accelerate both oxidation and polymer formation. This underscores the importance of characterizing oils not merely by their fatty acid percentages, but by the specific molecular architecture that dictates reactivity under thermal stress. Oxidative degradation, while often viewed solely as a detrimental process, also plays a functional role in the development of fried food characteristics, such as aroma and flavor. However, the same reactions that generate desirable sensory attributes can also produce harmful by‐products, including trans fatty acids, cyclic monomers, and volatile carbonyls. The dual nature of lipid oxidation in frying systems necessitates a more nuanced approach to process control—one that balances sensory quality with toxicological safety.

Analytical approaches to monitor oil degradation have expanded significantly. Conventional indices such as peroxide value, anisidine value, and total polar materials offer useful benchmarks but lack specificity and responsiveness under the fast‐changing conditions of commercial frying. The application of advanced spectroscopic and chromatographic techniques, including BBCEAS, GC–MS, and HPLC‐DAD, enables the real‐time tracking of degradation pathways with greater precision. These methods not only provide more accurate assessments of oil quality but also support the identification of emerging markers for early‐stage oxidation and polymerization. Thermal stability is further modulated by minor oil constituents, including tocopherols, phytosterols, and phenolic compounds. These substances can act synergistically or antagonistically with one another, depending on their concentration, structure, and interaction with metal ions or moisture. For example, certain terpenes exhibit antioxidant activity under frying conditions but may degrade into pro‐oxidant intermediates when exposed to prolonged heat. Understanding these interdependencies is essential for formulating oils with tailored oxidative behaviors suited to specific frying applications.

From a process engineering perspective, factors such as fryer design, surface‐to‐volume ratio, oxygen exposure, and oil turnover rate all play pivotal roles in modulating degradation kinetics. The presence of surface‐active compounds and food moisture contribute to foam formation and oxygen diffusion, which further exacerbate oxidation. The use of defoamers and oil replenishment protocols, while often considered secondary, can have a substantial cumulative effect on oil longevity and quality. In the context of food safety and regulation, there is growing attention to cumulative exposure to lipid‐derived degradation products. Regulatory frameworks in various countries now impose limits on FFAs, total polar compounds, and specific aldehydes, yet discrepancies in threshold values and testing protocols remain. More standardized approaches are needed to harmonize quality control across international food systems.

Looking ahead, innovation in frying technology—such as vacuum frying, air frying, and the use of structured emulsions—presents new opportunities to reduce oil degradation while preserving the sensory quality of fried foods. However, these systems introduce new variables in heat and mass transfer, necessitating further research into how they affect lipid chemistry, nutrient retention, and consumer acceptability.

## 8 Conclusion

This work reviewed the chemical changes occurring in deep‐fat frying, focusing on reaction mechanisms, oil degradation, and health implications. Three primary reaction pathways—homolytic, heterolytic, and concerted—drive oxidation, hydrolysis, and polymerization, leading to the formation of harmful compounds such as trans fats, acrylamide, and polymerized lipids. Factors influencing oil degradation include frying temperature, fatty acid composition, moisture content, and trace metals. Advanced analytical techniques such as GC, HPLC, and BBCEAS provide more accurate assessments of oil quality compared to conventional methods. Strategies to mitigate oil degradation include using stable oils, controlling frying conditions, replenishing oil, and incorporating antioxidants. Alternative frying techniques such as air frying and vacuum frying show promise in reducing toxic by‐products while maintaining food quality. Regulatory measures and consumer awareness are crucial for minimizing health risks associated with deep‐fat frying. Further research is needed to develop innovative frying technologies and improve oil stabilization strategies for safer, healthier fried foods.

## Author Contributions

**Tariq Ahmed:** methodology (equal), writing – review and editing (equal). **Mostafa Almdaaf:** writing – review and editing (equal). **Husayn Mohammed Omar Abu Hallalah:** writing – review and editing (equal). **Shamsudeen Jibia:** writing – review and editing (equal). **Naser Bazina:** conceptualisation, methodology, validation, formal analysis, investigation, data curation, project administration and funding acuisition.

## Ethics Statement

The authors have nothing to report.

## Conflicts of Interest

The authors declare no conflicts of interest.

## Data Availability

This review article does not involve the generation or analysis of new datasets. All data discussed is derived from previously published sources, which are cited appropriately throughout the manuscript. Readers can access the original data through the referenced publications.
